# Detection of germline *BRCA1* mutations by Multiple-Dye Cleavase Fragment Length Polymorphism (MD-CFLP) method

**DOI:** 10.1054/bjoc.2001.1988

**Published:** 2001-09

**Authors:** S Casadei, L Cortesi, V Pensotti, P Radice, M Pierotti, D Amadori, D Calistri

**Affiliations:** Istituto Oncologico Romagnolo, Forlì, 47100, Italy; Department of Medical Oncology, Morgagni-Pierantoni Hospital, Forlì, 47100, Italy; Istituto Nazionale Tumori, Milano, 20133, Italy

**Keywords:** mutational analysis, hereditary breast cancer, *BRCA1*, CFLP

## Abstract

We describe the Multiple-Dye Cleavase Fragment Length Polymorphism (MD-CFLP) method set up for a sensitive and preliminary rapid screening of *BRCA1* mutations. We analysed exons 11 and 16, which are known to cover slightly more than 70% of the whole coding region of the gene, subdivided into 4 amplicons and labelled with different fluorescent dUTPs. MD-CFLP was first utilised on a panel of 30 DNA samples in which the presence of single-base substitutions or small deletions/insertions had been previously identified by direct sequencing as gold standard, in order to define the optimal conditions in terms of PCR amplification and temperature of digestion. In a second step, we blindly analysed 21 DNA samples by MD-CFLP to verify its reliability. The sensitivity and specificity of MD-CFLP were both 100% in the first study, and 80% and 94%, respectively, in the blind sample assay. Our results demonstrate the capability of the MD-CFLP method to detect DNA sequence alterations in fragments of more than 1 kb. We conclude that CFLP is a powerful tool in mutational analysis, offering reliable results in a shorter time and at a lower cost than conventional methods, and its potential can be enhanced when internal fluorescent labelling and laser detection are used. © 2001 Cancer Research Campaignhttp://www.bjcancer.com


British Journal of Cancer (2001) 85(6), 845–849
© 2001 Cancer Research Campaign
doi: 10.1054/ bjoc.2001.1988, available online at http://www.idealibrary.com on IDEAL

### Detection of germline *BRCA1* mutations by Multiple-Dye Cleavase Fragment Length Polymorphism (MD-CFLP) method

**S Casadei[1], L Cortesi[1], V Pensotti[3], P Radice[3], M Pierotti[3], D Amadori[2] and D Calistri[1]**

[1]Istituto Oncologico Romagnolo, 47100 Forlì, Italy; [2]Department of Medical Oncology, Morgagni-Pierantoni Hospital, 47100 Forlì, Italy; [3]Istituto Nazionale Tumori, 20133 Milano, Italy

**Summary** We describe the Multiple-Dye Cleavase Fragment Length Polymorphism (MD-CFLP) method set up for a sensitive and preliminary rapid screening of *BRCA1* mutations. We analysed exons 11 and 16, which are known to cover slightly more than 70% of the whole coding region of the gene, subdivided into 4 amplicons and labelled with different fluorescent dUTPs. MD-CFLP was first utilised on a panel of 30 DNA samples in which the presence of single-base substitutions or small deletions/insertions had been previously identified by direct sequencing as gold standard, in order to define the optimal conditions in terms of PCR amplification and temperature of digestion. In a second step, we blindly analysed 21 DNA samples by MD-CFLP to verify its reliability. The sensitivity and specificity of MD-CFLP were both 100% in the first study, and 80% and 94%, respectively, in the blind sample assay. Our results demonstrate the capability of the MD-CFLP method to detect DNA sequence alterations in fragments of more than 1 kb. We conclude that CFLP is a powerful tool in mutational analysis, offering reliable results in a shorter time and at a lower cost than conventional methods, and its potential can be enhanced when internal fluorescent labelling and laser detection are used. © 2001 Cancer Research Campaign http://www.bjcancer.com

**Keywords**: mutational analysis; hereditary breast cancer; *BRCA1*; CFLP

For breast cancer hereditary syndrome, 2 major susceptibility genes have been cloned, *BRCA1* (Miki et al, 1994) and *BRCA2* (Wooster et al, 1995; Tavtigian et al, 1996), and additional but as yet unidentified genes are suspected to be involved (Serova et al, 1997). The contribution of mutations in *BRCA1* and *BRCA2* genes to the population incidence of inherited breast cancer has been described (Shattuck-Eidens et al, 1997; Ford et al, 1998; Hartge et al, 1999), yet estimates are still inconclusive due to the complexity of analysis of large-sized genes without hot-spots for sequence variation and to the substantial number of samples required to obtain reliable estimates. Intensive mutation analyses have been conducted on *BRCA* genes using different techniques and have led to the identification of more than 200 mutations, scattered throughout the genes. Complete direct sequencing assures the highest sensitivity, but is laborious and time-consuming, also considering the genomic structure and size of the genes, so that less sensitive pre-screening scanning methods, such as SSCP (Friedman et al, 1994), DGGE (Stoppa-Lyonnet et al, 1997), and PTT (Hogervorst et al, 1995) are commonly used.

In order to provide a more feasible and less expensive test for mutation screening prior to sequencing, we evaluated the potential of Cleavase Fragment Length Polymorphism method (CFLP; Third Wave Technologies, Madison, WI), reported as capable of detecting single and multiple base alterations in fragments up to 2 kb in length (Brow et al, 1996). CFLP scanning method uses an endonuclease-based approach to detect sequence alterations in DNA samples. An engineered structure-specific endonuclease, the Cleavase I enzyme, recognises and cleaves the 5′ end of secondary structures (i.e. stem-loops) that form when thermally denatured DNA strands are cooled, at the junction between single-stranded and double-stranded DNA. Digestion results in a collection of fragments that is unique to a specific DNA strand. Changes in the sequence of that strand, such as single point mutations, insertions, or deletions, will alter the secondary structures formed and the CFLP pattern detected. To date the CFLP method has been used in the differentiation of microbial species (Brow et al, 1996), hepatitis C virus genotyping (Marshall et al, 1997; Sreevatsan et al, 1998) and has been offered as an alternative to SSCP (Maddox et al, 1997; Rosetti et al, 1997), HA (Rosetti et al, 1997) and DGGE (Eisinger et al, 1998) analysis for mutation scanning.

We developed a DNA analysis procedure based on fluorescent dNTP incorporation, Cleavase I endonuclease digestion and simultaneous, multiple fragment detection, by combining the CFLP procedure with the GeneScan software, and called it Multiple-Dye CFLP (MD-CFLP). By incorporating different internal labelling and allowing for the size of the test fragment, it is possible to analyse patterns corresponding to the four different *BRCA1* amplicons loaded on a single lane of a denaturing polyacrylamide gel.

*Received 5 February 2001*
*Revised 14 May 2001*
*Accepted 15 May 2001*

*Correspondence to:* D Calistri

### MATERIAL AND METHODS

### Case series

We analysed a first sample panel of 30 patients with familial breast and/or ovarian cancer. 27 samples came from patients enrolled in an international multicentric study involving institutions from Italy, Finland, Germany, Switzerland and the United States

(Shattuck-Eidens et al, 1997) and were provided by the Division of Medical Oncology/Pierantoni Hospital (Forlì, Italy). 3 samples were provided by the 'Istituto Nazionale Tumori' (INT; Milan, Italy).

A second sample panel of 21 patients was further used for blind MD-CFLP analysis and was provided by the 'Istituto Nazionale Tumori' (INT; Milan, Italy).

### DNA preparation and PCR amplification

DNA was isolated from peripheral blood lymphocytes by standard phenol/chloroform procedure. Exons 11 and 16 of *BRCA1* were PCR-amplified from samples containing a sequence alteration, mutation or polymorphism within each exon, as well as from wild-type samples. Exon 11, which spans over half of the gene (3426 bp), was divided into 3 partially overlapping fragments, including flanking intronic regions, for MD-CFLP analysis (Table 1): fragment A, 1399 bp, spanning the flanking upstream intron and the 5′-terminus of exon 11; fragment B, 1221 bp, covering the central portion of the exon; and fragment C, spanning the 3′-terminus and the flanking downstream intron. Exon 16, with its flanking intronic regions, 415 bp, was amplified as a single fragment due to its relatively small dimension. PCR mixtures contained 100 ng of DNA, 2 U of Takara ex Taq polymerase (Takara Shuzo, Co., Shiga, Japan), 0.2 mM of each dNTP, 10 pmol of each primer (Table 1), and 0.5 μM [R110] or [R6G] dUTP or 2 μM [TAMRA] dUTP (Perkin-Elmer, Foster City, CA) in 50 μl of 2 mM Tris-HCl (pH 8.0), 10 mM KCl, 2 mM $Mg^{2+}$, 0.01 mM EDTA, 0.1 mM DTT, 0.05% Tween-20, 0.05% Nonidet P-40, 5% glycerol. After initial denaturation for 2 min at 94˚C, 40 cycles were performed with denaturation at 94˚C for 1 min, annealing at 58˚C for 90 s, and extension at 72˚C for 1 min. A final extension step of 5 min at 72˚C was added. The fragments were labelled by incorporation of distinct fluorescent dUTPs during PCR amplification. Labelling was always performed using the same dye with respect to a specific fragment to avoid variability in DNA structure due to incorporation of fluorescent dUTP instead of the normal thymidine. PCR products were precipitated in 2 M ammonium acetate and isopropanol for 10 min at room temperature and washed with 70% ethanol to remove unincorporated primers, labelled dUTP and salts that could affect Cleavase I activity and double-strand - reannealing. Better yields were obtained adding 1 μl (20 μg) of glycogen prior to precipitation (Boehringer Mannheim). Dried pellets were resuspended in 20 μl of sterile, distilled water. Concentration of PCR products was estimated by agarose gel electrophoresis and ethidium bromide staining.

### MD-CFLP analysis

For each MD-CFLP reaction, 200 – 400 ng of internally labelled PCR product were diluted in distilled water in a final reaction volume of 20 μl. Samples were heat-denatured for 30 s at 94˚C, quickly cooled down to the amplicon-specific reaction temperatures (Table 1) to allow secondary structure formation, and mixed with preheated enzyme mixture containing 25 U of Cleavase I enzyme (Third Wave Technologies, Madison, WI) and 0.25 mM $MnCl_2$ in 5.5 μl of 10 mM MOPS (pH 7.5), 0.05% Tween-20, 0.05% Nonidet P-40. Digestion temperatures for each amplicon were optimised at the values reported in Table 1 at scalar temperatures ranging from 40˚C to 60˚C by 5˚C or 2.5˚C increments. The digestion by Cleavase I was allowed for 15 min and stopped by the addition of 10 μl stop solution (95% deionised formamide, 10 mM EDTA and 0.05% blue dextran; SIGMA). Aliquots of the cleavage reaction were loaded onto a 5% w/v denaturing polyacrylamide gel (acrilamyde:bisaclylamide, 19:1, 7 M urea, 1 × TBE). Electrophoresis was carried out on an Applied Biosystems 373A DNA Sequencer (Applied Biosystems, Foster City, CA) equipped with GeneScan 672 Collection and Analysis software, and filter set A. Electrophoresis conditions were 560 V, 20 mA and 30 W constant power at a constant temperature of 40˚C for 12 hours.

### DNA sequence analysis

After a second independent CFLP analysis, samples exhibiting altered MD-CFLP patterns were confirmed by and sequenced on an Applied Biosystem 373A DNA Sequencer using internal primers and PRISM Dye Terminator Cycle Sequencing kit (Perkin-Elmer) according to the manufacturer's instructions.

### RESULTS

### Overview of MD-CFLP and multiplex array

We used Cleavase I digestion to generate a collection of fragments representing the unique DNA fingerprints of the test samples. We selected test samples harbouring small changes in the DNA sequence such as single-point mutations, insertions or deletions to be compared with wild-type samples. We combined Cleavase I digestion with differential internal labelling by fluorescent dUTP incorporation, and a simultaneous, multiple fragment detection by means of GeneScan software. The target sequence was amplified in a single PCR reaction containing an optimised dNTP mix and a limiting amount of fluorescent dUTP and digested by Cleavase I enzyme. Optimal conditions were defined by comparison of

**Table 1** *BRCA1* primers and conditions for multiple-dye CFLP reactions

| Fragment | Primer | Primer sequence (5′ → 3′) | Size (bp) | Dye (dUTP) | Cleavase reaction |
|---|---|---|---|---|---|
| exon 11 | | | | | |
| A | BR1 E11A 5′ | CCT CCA AGG TGT ATG AAG TA | 1399 | [R110] | 50˚C/15 min |
| | BR1 E11A 3′ | TGC TGT GCC TGA CTG GCA TT | | | |
| B | BR1 E11B 5′ | GCG CTT GAA CTA GTA GTC AG | 1221 | [R6G] | 55˚C/15 min |
| | BR1 E11B 3′ | ACG GCT AAT TGT GCT CAC TG | | | |
| C | BR1 E11C 5′ | GGA ACA TTC AAT GTC ACC TG | 1122 | [TAMRA] | 52.5˚C/15 min |
| | BR1 E11C 3′ | AAT AGA CTG GGG CAA ACA CA | | | |
| exon 16 | | | | | |
| | BR1 E16 5′ | ACA GAG ACC AGA ACT TTG TA | 415 | [TAMRA] | 42.5˚C/15 min |
| | BR1 E16 3′ | CTT AGT CAT TAG GGA GAT AC | | | |

 © 2001 Cancer Research Campaign wild-type and altered patterns of corresponding fragments with regard to good pattern complexity and even distribution of peaks, avoiding sample over-digestion. Once the best MD-CFLP conditions were established for each amplicon and the pattern reproducibility verified, the banding pattern resulting from Cleavase digestion was visualised on an Applied Biosystems 373A DNA Sequencer. By adapting custom matrices to GeneScan software, we accurately attributed signal intensity to specific fluorochromes and subtracted the background noise caused by interfering signals. Multiple loading was determined according to minimal overlapping between migration distances of amplicons labelled with the same dye.

MD-CFLP patterns of the 4 differently labelled BRCA1 amplicons were analysed in single and multiplex array. Comparison of the resulting electropherograms showed no interference between differently labelled samples loaded onto the same lane with respect to single-lane loaded samples on the same gel (data not shown).

### DNA sequencing of wild-type and mutated *BRCA1* samples

A complete sequence analysis of the *BRCA1* coding sequence and flanking intronic regions was performed on 30 DNA samples as described previously. 35 amplicons were developed for *BRCA1* sequencing. 12 sequence variants were detected in exons 11 and 16 including 3 deleterious mutations, 2 variants of uncertain significance and 7 common polymorphisms. The 3 deleterious alterations were frame-shift mutations due to single- or double-nucleotide insertions/deletions. One of the two uncertain variants identified, corresponding to a base substitution in the intronic region between exons 11 and 12, had never been reported before. Polymorphic sites 2201C/T, 2430T/C, P871L, E1038G, and K1138R in exon 11 and S1613G in exon 16 were associated in 11 (36%) of the samples analysed, giving a wild-type phenotype. Polymorphism Q356R was independently detected in 2 samples. None of the mutations or unclassified variants were associated with polymorphisms in this sample panel.

### MD-CFLP analysis of *BRCA1* samples

Of the 120 PCR products analysed by the MD-CFLP method (4 fragments for each of the 30 samples), 73 contained sequence alterations. Corresponding sequencing data are reported. Three deleterious mutations, 2 uncertain variants, and 68 common polymorphisms were identified (Table 2). Thus, both sensitivity and specificity of MD-CFLP analysis in detecting mutations were 100%. Samples harbouring sequence alterations gave rise to unique reproducible fingerprints easily distinguishable from the corresponding wild-type and identifiable as variants. Polymorphisms were always associated with reproducible peaks in the digestion patterns. Representative electropherograms are reported in Figure 1. Complete electropherograms representing exon 11 and exon 16 digestion patterns of 2 samples harbouring sequence alterations and a sample exhibiting the wild-type sequence, as they result from multiple fragment analysis, are illustrated in Figure 1A.

Exon 11/fragment B detailed electropherogram of a sample containing 3 single-nucleotide polymorphisms, compared with the corresponding wild-type sequence, is illustrated in Figure 1B. PCR-amplified fragments were internally labelled with R6G fluorescent dye. Because of the internal labelling, we were able to detect all possible products that originated from the digestion. Since Cleavase I digestion is sequence-specific, the appearance or disappearance of peaks in the MD-CFLP pattern revealed the presence of alterations in the given sequence compared to the wild-type. Major deviations from the standard pattern were detected in the central portion of the electropherogram, where 3 novel peaks rose up. A reduction or increase in peak intensities was observed in at least 3 sites along the pattern.

Exon 11/fragment C MD-CFLP pattern from a sample with a base substitution in the intron downstream exon 11 compared with a wild-type sequence is shown in Figure 1C. PCR-amplified fragments were internally labelled with TAMRA fluorescent dye. In this instance, the sequences of the mutated and wild-type fragments differed only in terms of a single nucleotide change, yet several changes were found in their fingerprints. Variation of intensity was evident in 3 peaks in the left portion of the electropherogram and in 1 in the right portion. These electropherograms highlighted a clear distinction between wild-type and altered samples.

### Sensitivity of MD-CFLP patterns

To assess the sensitivity of the fingerprints originated by MD-CFLP, we examined a random panel of 21 DNA samples kindly

**Table 2** *BRCA1* mutations and common polymorphisms utilised for multiple-dye CFLP set-up

| Fragment | Designation[a] | Sequence modification | Type of alteration[b] | Number of samples |
|---|---|---|---|---|
| 11A | 916delTT | delete TT | F | 1 |
| 11A | Q356R | A→G | P | 2 |
| 11A | 1499insA | insert A | F | 1 |
| 11A | S645Y | C→A | UV | 1 |
| 11B | 2201C/T | C→T | P | 11 |
| 11B | 2430T/C | T→C | P | 11 |
| 11B | P871L | C→T | P | 11 |
| 11C | E1038G | A→G | P | 11 |
| 11C | 3345delAG | delete AG | F | 1 |
| 11C | K1183R | A→G | P | 11 |
| 11C | IVS11+8[c] | A→G | UV | 1 |
| 16 | S1613G | A→G | P | 11 |

[a]Designations as reported in the Breast Cancer Information Core database (BIC) at www.nhgri.nih.gov/Intramural_research/Lab_transfer/Bic. [b]F = frameshift mutation; P = polymorphism; UV = unclassified variant. [c]Not previously reported.

© 2001 Cancer Research Campaign 

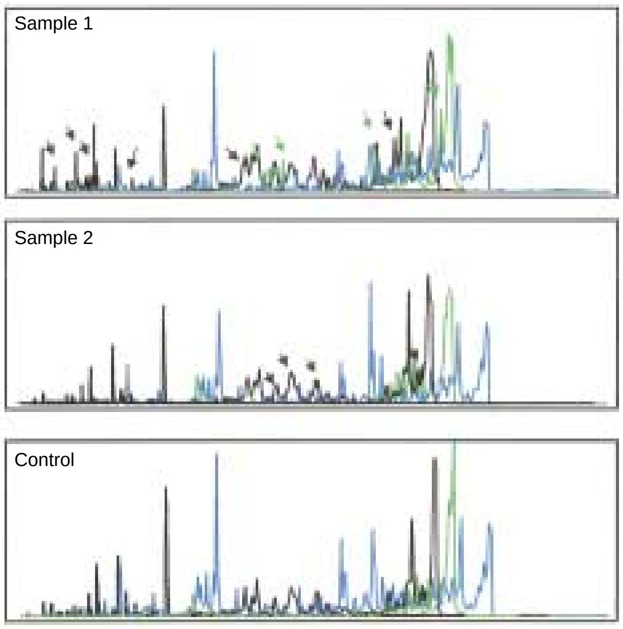

**Figure 1A** MD-CFLP complete electropherograms. Sample 1: presence of polymorphisms in exon 16 (S1613G), and in exon 11 fragment B (2201C/T, 2430T/C, P871L) and C (E1038G, K1183R). Sample 2: sequence variant due to a base substitution in the intronic region downstream exon 11. Control: electropherogram of a wild-type sequence. Digestion patterns corresponding to fragments A (blue), B (green), and C (black) of exon 11 and exon 16 (black) are represented and sites of pattern variations are indicated with coloured arrows. All fragments were labelled as reported in Section 2.2

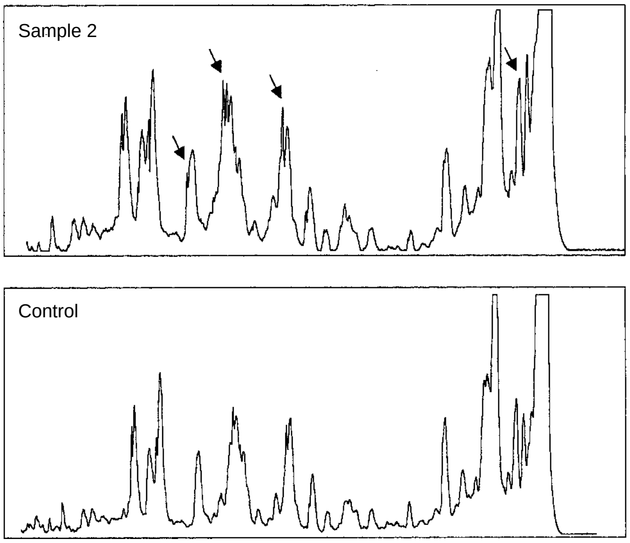

**Figure 1C** Electropherogram details of exon 11 fragment C. Comparison between sample 2 carrying a base substitution in the intronic region (IVS11+8) and the control. Main pattern variations are indicated by arrows

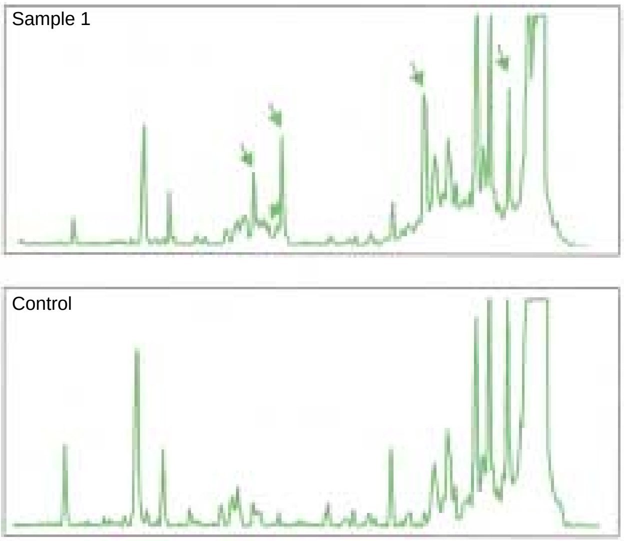

**Figure 1B** Electropherogram details exon 11 fragment B. Comparison between sample 1 harbouring three common polymorphisms (2201C/T, 2430T/C, P871L) and the control. Main differences are indicated with arrows provided by the INT (Milan, Italy) whose sequence characterisation was unknown to us. By means of MD-CFLP analysis and further ascertainment by direct sequencing we were able to correctly characterise 79 of the 84 fragments analysed and 8 of the 10 mutations present in the sample panel. Prior to sequencing, each sample was submitted to 2 MD-CFLP analyses: a 100% consistency was observed. The 84 PCR products were screened by the MD-CFLP method (4 fragments for each of the 21 samples), as described earlier. Of the 13 rare alterations identified, 8 corresponded to known variants and 5 did not correspond to any alteration of the test sequences: 4 alterations in fragment A, 1 in fragment B, 3 in fragment C of exon 11 were correctly identified; 2 alterations in fragment A, 2 in fragment C of exon 11 and 1 in exon 16 turned out to be false positives (Table 3). Fragments showing altered MD-CFLP patterns were verified by direct sequencing. The specificity of the MD-CFLP analysis in this blind sample panel was 94%. We further compared our final data with the sample characterisation reported by the INT and found that 2 mutations consisting of single-base substitutions in exon 11/fragment B and fragment C had been missed, namely the missense mutations R841W and 3159A/G. Thus, the sensitivity of the MD-CFLP analysis in this sample panel was 80%.

Common sequence variants corresponding to polymorphisms were identified according to pattern variation frequency in 9 of the 21 samples in the panel. Polymorphisms 2201C/T, 2430T/C, P871L, E1038G and K1183R were characterised with 100% sensitivity by subsequent sequence investigation. Moreover, in 5 cases, polymorphisms were associated with rare sequence variants (1100delAT, 1221G > T, 1806C >T, 3159A > G and 3372insA).

### DISCUSSION

Recent findings in the field of cancer genetics have opened the way to molecular tests to identify high-risk patients carrying a mutation in a cancer-predisposing gene. The use of these tests is still controversial for ethical, psychological and social reasons, in addition to their still undefined role in prevention and early diagnosis strategies (Ponder, 1997). However, it is becoming increasingly important to develop methods of analysis that are highly sensitive, have a high throughput, and are specific and accessible in terms of time and costs (Sidransky, 1997). An accurate definition of their reliability and limits is thus necessary in order to correctly evaluate the laboratory data impact on global patient management.

Although sequencing technology has seen a marked improvement in recent years in terms of throughput and reliability, it

© 2001 Cancer Research Campaign

**Table 3** *BRCA1* blind samples analysed by multiple-dye CFLP

| Sample | Analysed fragments | | | | Sequence modification | Designation[a] | Altered fragment |
|---|---|---|---|---|---|---|---|
| | 11A | 11B | 11C | 16 | | | |
| 1 | + | – | – | – | delete AT | 1100delAT | 11A |
| 2 | + | – | – | – | delete A | 1206delA | 11A |
| 3 | + | – | – | – | G→T | 1221G > T[b] | 11A |
| 4 | + | – | – | – | C→T | Q563X | 11A |
| 5 | – | + | – | – | delete TCAA | 2846del4 | 11B |
| 6 | – | – | + | – | insert A | 3373insA | 11C |
| 7 | – | – | + | – | C→T | 3522C > T[b] | 11C |
| 8 | – | – | + | – | G→T | 4023G > T[b] | 11C |
| 9 | – | – | + | – | C→T | R841W | 11B |
| 10 | – | – | – | + | A→G | 3159A > G[b] | 11C |
| 11 | + | – | – | – | none | wt | none |
| 12 | + | – | – | – | none | wt | none |
| 13 | – | – | + | – | none | wt | none |

[a]Designations as reported in the Breast Cancer Information Core database (BIC) at ww.nhgri.nih.gov/Intramural_research/Lab_transfer/Bic. [b]Not previously reported.

remains too cumbersome and expensive for large-sized genes where different mutations are scattered throughout the sequence. A good scanning technique should be much cheaper and less labour-intensive than sequencing. There is not one technique that is overwhelmingly preferred over the others. Factors that we considered in the selection of the MD-CFLP mutation scanning technique are as follows: (i) the size of the gene of interest; (ii) the high throughput vs low throughput; (iii) the degree of sensitivity and specificity required; and (iv) the cost of and time required for analysis.

We developed a DNA analysis procedure based on fluorescent dNTP incorporation, Cleavase I endonuclease digestion and simultaneous, multiple fragment detection, which we called Multiple-Dye CFLP. We defined the best conditions in terms of PCR amplification and temperature of digestion for a rapid prescreening mutation analysis capable of ensuring a sensitivity and specificity higher than 90%. Internal labelling increases the sensitivity of the method and hence the detection of all the reaction products is possible despite their size and relative intensity. This approach allows a simultaneous check of both strands without the need for separate single-strand reactions, which is important because some alterations can affect the MD-CFLP pattern of one strand more than that of the complementary one (Brow et al, 1996). The availability of GeneScan software permits the analysis of complex electropherograms originated by internal labelling. Thus, Multiple-Dye CFLP enables the simultaneous analysis of nearly 70% of *BRCAI* in a single lane of a polyacrylamide gel and speeds up the detection of sequence alterations in large numbers of DNA samples. Moreover, polymorphisms can be recognised on the basis of specific fingerprints, thus limiting sequence characterisation to variants of pathological or still unknown significance. For these reasons we are confident that this approach will be of impact in genetic testing for cancer susceptibility, especially when several genes are to be investigated in one syndrome.

### ACKNOWLEDGEMENTS

The authors wish to thank Professor Rosella Silvestrini for her invaluable scientific contribution and Ms Grainne Tierney for editing the manuscript. This work was supported by a grant from Istituto Oncologico Romagnolo.

### REFERENCES

Brow MAD, Oldenburg MC, Lyamichev V et al (1996) Differentiation of bacterial 16S rRNA genes and intergenic regions and *Mycobacterium tuberculosis* katG genes by structure-specific endonuclease cleavage. *J Clin Microbiol* **34**: 3129–3137

Eisinger F, Jacquemier J, Charpin C, Stoppa-Lyonet D et al (1998) Mutations at *BRCA1*: the medullary breast carcinoma revisited. *Cancer Res* **58**: 1588–1592

Ford D, Easton DF, Stratton M and the Breast cancer Linkage Consortium (1998) Genetic heterogeneity and penetrance analysis of the *BRCA1* and *BRCA2* genes in breast cancer families. *Am J Hum Genet* **62**: 676–689

Friedman LS, Ostermeyer EA, Szabo CI et al (1994) Confirmation of *BRCA1* by analysis of germline mutations linked to breast and ovarian cancer in ten families. *Nature Genet* **8**: 399–404

Hartge P, Struewing JP, Wacholder S, Brody LC and Tucker MA (1999) The prevalence of common *BRCA1* and *BRCA2* mutations among Ashkenazi Jews. *Am J Hum Genet* **64**: 963–970

Hogervorst FB, Cornelis RS, Bout M et al (1995) Rapid detection of *BRCA1* mutations by the protein truncation test. *Nature Genet* **10**: 208–212

Maddox LO, Li P, Bennett A, Descartes M and Thompson JN (1997) Comparison of SSCP analysis and CFLP analysis for mutation detection in the human iduronate 2-sulfatase gene. *Biochem Mol Biol Int* **43**: 1163–1171

Marshall DJ, Heisler LM, Lyamichev V et al (1997) Determination of hepatitis C virus genotypes in the United States by cleavase fragment length polymorphism analysis. *J Clin Microbiol* **35**: 3156–3162

Miki Y, Swensen J, Shattuck-Eidens D et al (1994) A strong candidate for the breast and ovarian cancer susceptibility gene BRCA1. *Science* **266**: 66–71

Ponder B (1997) Genetic testing for cancer risk. *Science* **278**: 1050–1054

Rosetti S, Englisch S, Bresin E, Pignatti PF and Turco AE (1997) Detection of mutations in human genes by a new rapid method: cleavage fragment length polymorphism analysis (CFLPA). *Mol Cell Probes* **11**: 155–160

Serova OM, Mazoyer S, Puget N et al (1997) Mutations in *BRCA1* and *BRCA2* in breast cancer families: are there more breast cancer-susceptibility genes? *Am J Hum Genet* **60**: 486–495

Shattuck-Eidens D, Oliphant A, McClure M et al (1997) *BRCA1* sequence analysis in women at high risk for susceptibility mutations. *JAMA* **278**: 1242–1250

Sidransky D (1997) Nucleic acid-based methods for the detection of cancer. *Science* **278**: 1054–1058

Sreevatsan S, Bookout JB, Ringpis FM et al (1998) Algorithmic approach to high-throughput molecular screening for alpha interferon-resistant genotypes in hepatitis C patients. *J Clin Microbiol* **36**: 1895–1901

Stoppa-Lyonnet D, Laurent-Puig P, Essioux L et al (1997) *BRCA1* sequence variations in 160 individuals referred to a breast/ovarian family cancer clinic. *Am J Hum Genet* **60**: 1021–1030

Tavtigian SV, Simard J, Rommens J et al (1996) The complete *BRCA2* gene, mutations in chromosome 13q-linked kindred. *Nature Genet* **12**: 333–337

Wooster R, Bignell G, Lancaster J et al (1995) Identification of the breast cancer susceptibility gene *BRCA2*. *Nature* **378**: 789–792

*© 2001 Cancer Research Campaign*
